# It happened to a friend of a friend: inaccurate source reporting in rumour diffusion

**DOI:** 10.1017/ehs.2020.53

**Published:** 2020-11-11

**Authors:** Sacha Altay, Nicolas Claidière, Hugo Mercier

**Affiliations:** 1 Institut Jean Nicod, Département d’études cognitives, ENS, EHESS, PSL University, CNRS, Paris, France; 2 Aix Marseille University, CNRS, LPC, FED3C, Marseille, France

**Keywords:** Rumours, rumors, source, cultural evolution, redundancy, reputation

## Abstract

People often attribute rumours to an individual in a knowledgeable position two steps removed from them (*a credible friend of a friend*), such as ‘my friend's father, who's a cop, told me about a serial killer in town’. Little is known about the influence of such attributions on rumour propagation, or how they are maintained when the rumour is transmitted. In four studies (*N =* 1824) participants exposed to a rumour and asked to transmit it overwhelmingly attributed it either to a credible friend of a friend, or to a generic friend (e.g. ‘a friend told me about a serial killer in town’). In both cases, participants engaged in *source shortening*: e.g. when told by a friend that ‘a friend told me …’ they shared the rumour as coming from ‘a friend’ instead of ‘a friend of friend’. Source shortening and reliance on credible sources boosted rumour propagation by increasing the rumours’ perceived plausibility and participants’ willingness to share them. Models show that, in linear transmission chains, the generic friend attribution dominates, but that allowing each individual to be exposed to the rumour from several sources enables the maintenance of the credible friend of a friend attribution.

## Graphical abstract

**Figure:**
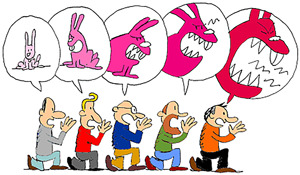

**Media summary:** Attribution to a credible friend of a friend boosts rumour propagation and remains stable as the rumour is transmitted.

## 1. Introduction

In the month of March 2020, as the epidemic of COVID-19 was starting to seriously hit the US, a rumour was widely disseminated:

Please be advised, within 48 to 72 Hours the president will evoke what is called the Stafford act. Just got off the phone with some of my military friends up in DC who just got out of a two hour briefing. The president will order a two week mandatory quarantine for the nation. Stock up on whatever you guys need to make sure you have a two week supply of everything. Please forward to your network. (Reuters, 2020)

This rumour was typically presented as coming from a friend of the individual passing it along, illustrating a common feature of inaccurate rumours and urban legends: the attribution to a friend (the military friend) of a friend (who passed it along). The dramatic (but false) events depicted are attested not by someone the speaker knows directly, but by someone known by someone the speaker knows. Moreover, the original source (the friend of the friend) is supposed to be credible either because they have witnessed or experienced some event themselves, or because they are in a position of authority (in the case above, because of their position in the military).

Attribution to a credible friend of a friend is widely thought to help rumours spread, in particular by making them more credible (Blake, McFaul & Porter,^1974^, p. 7; DiFonzo & Bordia, 2007, p. 101). For example, Knapp (1944) compiled and catalogued more than a thousand World War II rumours and found that successful rumours tended to be attributed to an authoritative source, giving it an ‘appearance of veracity’ (p. 30). This article investigates (a) how people report and modify sources when transmitting a rumour, (b) whether sourcing influences how plausible a rumour is and how willing participants are to share it, and (c) under which conditions attributions to a credible friend of a friend can be maintained across long transmission chains. Before coming back to these questions, we offer a brief primer on rumour transmission, stressing the role of source reporting.

### 1.1. Source reporting and rumour transmission

Whenever people are not entirely satisfied with official channels such as government communication and mainstream media, rumours flourish (Allport & Postman, 1947; Shibutani, 1966). In some contexts, the accuracy of rumours has been shown to be extremely low. This is true for instance in the aftermath of a natural disaster, as inflated rumours about the damages spread (Diggory, 1956; Prasad, 1935; Sinha, 1952), but also in the context of inter-ethnic violence (Horowitz, 2001). In other contexts, however, rumours are overwhelmingly accurate (for a review, see, DiFonzo & Bordia, 2007). For instance, studies have shown that rumours circulating in small groups of soldiers (Caplow, 1947; Walton, 1961) or coworkers (Davis, 1972; Marting, 1969; Rudolph, 1973) about their professional life are nearly always true.

One of the factors that distinguish the spread of accurate and inaccurate rumours is the accuracy of the sourcing: when a rumour is transmitted, is its actual source accurately portrayed? Accurate rumours are accompanied by precise sources, such as ‘John told me that Mary was going to be sacked’ (Arndt, 1967, p. 66, cited by DiFonzo & Bordia, 2007, p. 166; Caplow, 1947). Given that such rumours circulate in groups of people who know each other, those who receive the rumour can evaluate its plausibility based on the expertise of the original source (here, John). Moreover, they can hold this original source accountable if the rumour turns out to be mistaken, which provides incentives for people not to start unfounded rumours.

In contrast, inaccurate rumours are most often accompanied by either vague (‘everybody says’) or inaccurate (‘it happened to a friend of a friend’) sourcing (e.g. Bonhomme, 2015). In such cases, holding the original source accountable is impossible, and the incentives for accuracy are reduced. For example, in 1969, rumours about Jewish retailers kidnapping young women in the fitting rooms of their stores spread in the small French town of Orléans. The researchers who recorded these rumours noted that they were often ascribed to a credible relation of a relation, such as: ‘a friend's father is a cop, and he's investigating a kidnapping case’. or ‘my cousin's wife is a nurse, and she treated the victim of an attempted kidnapping’ (E. Morin, 1969, p. 113). This pattern of attributing an event to a credible source one step removed from the speaker – a credible friend of a friend – is a common feature in the spread of false rumours and urban legends (Heath et al., 2005; Kapferer, 1992, 2013; Nicolini, 1989; Turner, 1987). Just like in the days of the Orléans’ rumour, such sourcing continues to be used today, potentially facilitating the spread of racists rumours exacerbating ethnic tensions, with examples easy to find on Twitter (e.g. Bocchi, 2019) or WhatsApp (e.g. Rea, 2020).

The most remarkable feature of the credible friend of a friend attribution is its persistence throughout the transmission chain of a rumour. If people accurately portrayed their sources, a rumour they heard from a friend, and attributed to ‘a credible friend of a friend’ should be transmitted as ‘a credible friend of a friend of a friend’, which should then be transmitted as ‘a credible friend a friend of a friend of a friend’ – or maybe cut short to ‘someone told me’, for convenience. Instead, people who transmit credible friend of a friend rumours tend to shorten the source chain so that the credible source stays two steps removed from the speaker (Blehr, 1974, p. 42, cited by Tangherlini, 1990, p. 374; Dégh & Vazsonyi, 1974). As Edgar Morin, who studied Orléans’ rumour, noted: ‘each new transmitter [of the rumour] suppresses the new link, and rebuilds a chain with only two or three links’ (E. Morin, 1969, p. 113). If rumours attributed to a credible friend of a friend are more plausible, this attribution should further facilitate their spread, as people are more inclined to pass on rumours deemed more plausible (Jaeger et al., 1980; Rosnow et al., 1986; Tree & Weldon, 2007).

### 1.2. The current studies

To better understand the influence of sourcing on rumour propagation, and more particularly attributions to a credible friend of a friend, we conducted a series of studies measuring (a) how plausible participants found rumours with various sources, (b) how willing they were to share the rumours and (c) how they reported and modified the source of a rumour when sharing it.

In Study 1 and 1′ participants were asked to evaluate the plausibility of rumours attributed to various sources. Two features of the sources were manipulated: (a) the number of links (e.g. a friend, or a friend of a friend); and (b) the credibility of the source (e.g. a friend, or a cop investigating the case).

In Studies 2 and 3 participants read a rumour and then had to share the rumour from memory, allowing us to measure how participants reported the source of a rumour when sharing it. Study 2 focuses on rumours attributed to a credible friend of a friend; Study 3 replicates Study 2 and introduces rumours attributed to a generic friend.

In Study 3 participants were also asked how willing they would be to share the rumours on a Likert scale, and were exposed to less plausible version of the rumours used in Studies 1 and 2. This allowed us to test whether participants preferred to share: (a) rumours with fewer links; (b) rumours with more plausible content; or (c) rumours attributed to credible sources.

Studies 1–3 are based on a single transmission episode; they explore how a participant acquires and transmits a rumour, but without modelling it is often difficult to extrapolate the results of these single-step experiments to the large number of transmission events that would naturally occur in rumour propagation (Boyd and Richerson, 1988; Cavalli-Sforza and Feldman, 1981). Many models of cultural evolution focus on the direct source of the cultural element – here, the individual transmitting the rumour (e.g. Boyd & Richerson, 2005). In contrast, we are interested in the transmission, along the chain, of a specific element of the content of the rumour: the source it is attributed to (in contrast with the individual transmitting the rumour). Moreover, we are also interested in constructive processes (how modifications occurring during communication favour some content over others) instead of preservative processes (how fidelity is secured via specialized mechanisms such as copying heuristics). For instance, our models take into account the possibility that participants introduce, of their own accord, a credible source in the attribution of the rumour. The models that best fit these requirements are evolutionary causal matrices (Claidière et al., 2014) which allow simulation of the long-term effects of the transformations occurring at each episode of transmission (for examples see Miton et al., 2015; for review, see Miton & Charbonneau, 2018).

We use the results of Studies 2 and 3 to build evolutionary causal matrices and model transformations in the sources rumours are attributed to. Since they are a straightforward extrapolation from our experimental data, used only to better understand the long-term outcome of the results, these models assume linear transmission chains: one participant passes the rumour to a single other participant, who only receives one rumour. As a result, as in many other transmission chain experiments, losses increase at each generation (through modifications introduced by memory and random drift) until most information is lost (see, e.g. Bartlett, 1932; Miton & Charbonneau, 2018). A core feature of social transmission, which counteracts these losses, is redundancy and repetition (O. Morin, 2015). In natural settings, rumours are heard multiple times, from a variety of sources (e.g. Bonhomme, 2015; Thomas, 2007). Mathematical models of cultural transmission have shown that repetition and redundancy reinforce transmission chains, and allow cultural items to stabilize over time in a population even in the absence of high transmission fidelity (Acerbi & Tennie, 2016; Enquist et al., 2010; Kempe et al., 2014). For example, learning from multiple sources (redundancy) appears necessary for the cumulative cultural evolution of technical skills (Derex et al., 2013; Muthukrishna et al., 2014). Similarly, stories with only one source individual (no redundancy) disappear after a few episodes of transmission owing to imperfect transmission, whereas stories with at least two source individuals stabilize in cultural chains as redundancy compensates for transmission errors (Eriksson & Coultas, 2012, see also: O. Morin, 2015). Accordingly, in Study 4 we incorporate redundancy to study how the attribution to a credible friend of a friend can stabilize.

## 2. Study 1

The first study investigates how sourcing, and particularly attributions to a credible friend of a friend, affects the perceived plausibility of rumours. We excluded sources lacking ecological validity, such as rumours with more than three links and rumours without a credible source and three links (i.e. ‘a friend of a friend of a friend told me …’).

### 2.1. Participants

A power analysis for two-tailed *t-*tests with an estimated effect size of *d* = 0.50 (corresponding to a medium effect size), an *α*-level of 5% and a power of 80%, suggested that we needed a minimum of 64 participants per condition. Since we have five conditions, we needed a minimum of 320 participants.

We recruited 506 online participants from the US, UK, and Ireland, using Prolific Academic, paid £0.2. Five participants who did not correctly answer the attention check were removed from analysis, leaving 501 participants (320 women, mean age (*M*_Age_) = 34.37, SD = 11.43).

### 2.2. Design and procedure

After they had agreed to a consent form, participants were presented with a short vignette describing a friend telling them of a rumour. The content of the rumour (serial killer, escaped bear, *E. coli* infection, contaminated water reservoir; see below for examples, and Electronic Supplementary Materials for full stimuli set), as well as the source to which the rumour was attributed were manipulated in a between-participants design. Rumours were attributed to one of six sources, that varied as a function of the number of links (from one to three, e.g. ‘a friend of a friend’ is a two links attribution), and the presence or absence of an initial credible source. Since rumours with three links and no credible source, and rumours with more than three links, appeared too artificial (i.e. to the best of our knowledge, they are never reported in the observational literature), we only used the five other conditions.

Participants were then asked to evaluate the rumour's plausibility by answering ‘Considering what your friend said, how sure are you that [a bear actually escaped from the zoo]?’ on a seven-point Likert scale (‘Not sure at all’, 1; ‘Somewhat sure’, 4; ‘Completely sure’, 7). Finally, participants completed an attention check (see Electronic Supplementary Materials, ESM), and filled in basic demographic information.

### 2.3. Materials

Here is an example of a rumour about a bear that escaped from the zoo, attributed to a credible friend:

*Imagine the following situation:*

Today, while shopping, you run into a friend who tells you:

‘I just learned that a bear escaped from the zoo today. My father, who is working at the zoo, told me. Be careful!’

[*Page break*]

Considering what your friend said, how sure are you that a bear actually escaped from the zoo?

[*Quick reminder of what their friend said*]

*Likert scale: (1) Note sure at all – (2) – (3) – (4) Somewhat sure – (5) – (6) – (7) Completely sure*

For this story, the alternative attributions were:

- ‘A friend told me’.
- ‘A friend of a friend told me’.
- ‘A friend, whose father is working at the zoo told me’.
- ‘A friend of a friend, whose father is working at the zoo told me’.

All the vignettes used in this article can be found in the ESM.

### 2.4. Results and discussion

All statistical analyses were conducted in R (v.3.6.0, R Core Team, 2017), using R Studio (v.1.1.419, RStudio Team, 2015). Throughout this article we used parametric tests because we had normal distributions of the residuals and did not violate statistical assumptions (switching to non-parametric tests would have reduce our statistical power). The confidence intervals (CI) reported are 95%. In the studies presented in this article, we report all measures, manipulations and exclusions, and sample size was determined before any data analysis. All *t*-tests reported are Welch's *t*-tests, and all chi-square tests reported are chi-square goodness of fit tests.

**Figure 1.**
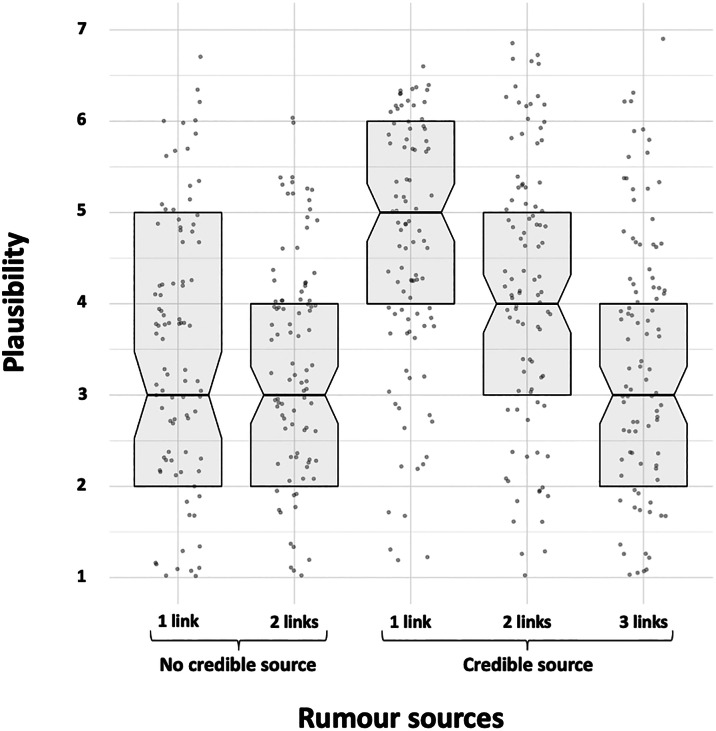
Boxplot of each rumour estimated plausibility depending on its source. The box represents the middle 50% of scores for the group, the line that divides the box is the median. The data points represent single answers.

We compared the plausibility of rumours attributed to a credible friend of a friend to the other attributions. Rumours attributed to a credible friend of a friend were deemed more plausible (*M* = 4.11; SD = 1.60) than rumours attributed to a friend (*M* = 3.43; SD = 1.66; *t*(196.88) = 2.94, *p* = 0.004, CI [0.23, 1.14], *d* = 0.42), to a friend of a friend (*M* = 3.28; SD = 1.40; *t*(193.90) = 3.89, *p* < 0.001, CI [0.41, 1.25], *d* = 0.55), to a credible friend of a friend of a friend (*M* = 3.33; SD = 1.52; *t*(196.29) = 3.52, *p* < 0.001, CI [0.34, 1.22], *d* = 0.50), but not a credible friend (*M* = 4.49; SD = 1.57; *t*(196.84) = −1.68, *p* = 0.09, CI [−0.82, 0.07], *d* = 0.24). See Figure 1 for a visual representaion of the results.

Notably, the credible friend of a friend attribution leads to more plausible rumours than its two most relevant alternatives: a friend who is also often observed in actual rumour transmission (i.e. ‘someone said’, ‘a friend said’), and a credible friend of a friend of a friend, which would be, for people receiving a rumour attributed to a credible friend of a friend, an accurate way of transmitting the rumour further.

Even if attributions to a credible friend (e.g. ‘My father, who is working at the zoo’) lead to more plausible rumours, such attributions are very rarely observed. To utter such a statement, people would have to lie blatantly to people who either already know better (i.e. the audience might know the speaker's father doesn't work at the zoo), or who might find out. The speaker sharing a rumour attributed to a credible friend would thus be producing an obvious lie for little expected benefit, something most people avoid doing (e.g. DePaulo et al., 1996).

## 3. Expriment 1′

To ensure the robustness of our findings, we replicated the most likely alternatives to attributions to a credible friend of a friend (i.e. a friend, and a credible friend of a friend of a friend). Design, procedure and materials are otherwise exactly the same as for Study 1.

### 3.1. Participants

A power analysis for a two-tailed *t*-test with an estimated effect size of *d* = 0.4 (corresponding to the smallest effect size of Study 1), an *α*-level of 5% and a power of 80% suggested that we needed a minimum of 100 participants per condition. Since we have three conditions, we needed a minimum of 300 participants.

We recruited 301 online participants from the US, UK and Ireland, using Prolific Academic, paid £0.2. Two participants who did not correctly answer the attention check were removed from analysis, leaving 299 participants (198 women, *M*_Age_ = 35.22, SD = 11.78).

### 3.2. Results and discussion

Rumours attributed to a credible friend of a friend (*M* = 3.68; SD = 1.70) were deemed slightly, but not significantly, more plausible than rumours attributed to a friend (*M* = 3.30; SD = 1.44; *t*(190.96) = 1.67, *p* = 0.10, CI [−0.07, 0.81], *d* = 0.24); they were also slightly, and significantly, deemed more plausible than rumours attributed to a credible friend of a friend of a friend (*M* = 3.19; SD = 1.55; *t*(195.76) = 2.12, *p* = 0.03, CI [0.03, 0.94], *d* = 0.30).

We ran fixed-effects meta-analysis model implemented in the ‘metafor’ R package (Viechtbauer, 2010) to aggregate the results of the two studies (giving more weight to effect sizes with the lowest standard errors). Across the two studies, rumours attributed to a credible friend of a friend were found more plausible than rumours attributed to a friend (*β* = 0.16 ± 0.072, *z* = 2.26, *p =* 0.02, CI [0.02, 0.30]) or to a credible friend of a friend of a friend (*β* = 0.20 ± 0.072, *z =* 2.74, *p =* 0.006, CI [0.06, 0.34]).

Studies 1 and 1′ confirm that, barring an obvious lie, the best way to make a rumour credible is to attribute it to a credible friend of a friend. However, for this plausibility boost to explain the success of rumours, the credible friend of a friend attribution must be preserved throughout the transmission chain, which is what we investigate in Study 2.

## 4. Study 2

In Study 2, participants were exposed to a rumour whose attribution varied (e.g. a credible friend, or a credible friend of a friend), and asked to transmit the rumour from memory. This study tests whether participants (accurately) introduce a new link in the attribution (e.g. turning ‘a credible friend of a friend’ into ‘a credible friend of a friend of a friend’), maintain the original attribution, or shorten it further to ‘a friend’. To extrapolate from the one transmission episode from the experiment, we build an evolutionary causal matrix that models what would happen if transmission episodes were repeated (on the importance of modelling to link experimental data to cultural trends, see, e.g. Boyd & Richerson, 2005; Kalish et al., 2007; Kirby et al., 2007).

### 4.1. Participants

A power analysis for a chi-square goodness of fit test with an estimated effect size of *w* = 0.30 (corresponding to a medium effect size), an *α*-level of 5% and a power of 80%, suggested that we needed a minimum of 88 participants per comparison. Since we intended toperform two comparisons, we needed a minimum of 176 participants.

We recruited 217 online participants from the US, UK and Ireland, using Prolific Academic, paid £0.3. We removed three participants who failed the attention check, seven who did not attempt to transmit the rumour, seven who explicitly refused to pass along the rumour, 11 who warned their friend but did not pass along the rumour (codes 1, 4 and 5 of our first coding), and six who did not provide any identifiable source (codes 0 and 2 of our second coding), leaving 184 participants (116 women, *M*_Age_ = 36.52, SD = 13.57).

### 4.2. Design and procedure

Participants saw one vignette from Study 1 with the rumour attributed a credible source. The number of links (one, a credible friend; two, a credible friend of a friend; or three, a credible friend of a friend of a friend) was manipulated between participants. Participants did not rate the plausibility of the rumour. Instead, on the next screen, they were asked to type what they would tell their best friend so as to warn them of the threat. Next, the participants were prompted by their best friend answering ‘Thank you for warning me! How do you know about this again?’ This second memorization task was designed to encourage participants to mention a source. When hearing a rumour in face-to-face conversations, people typically ask for a clarification or a source (Buckner 1965).

### 4.3. Materials

Here is an example of a rumour about a serial killer, attributed to a credible friend of a friend:

Imagine that you're doing grocery shopping and you run into a childhood friend who tells you:

‘I just learned that there's a serial killer in town. A friend, whose father is a cop investigating the case, told me. Be careful!’

[*Page break*]

Once you get home you decide to warn your best friend. You call them and tell them:

[*Free text entry*]

[*Page break*]

Your best friend says:

‘Thank you for warning me! How do you know about this again?’

What do you tell them?

[*Free text entry*]

### 4.4. Coding

The rumours transmitted by participants, as well as the source they were attributed to, were coded by one of the experimenters. The goal of the first code was to ascertain whether participants were reporting the rumour at all. The content of the rumours (first memorization task only) was coded as follows, the main goal of the coding being to remove participants who did not transmit the rumour (i.e. codes 1, 4 and 5):

- 1 = warning without any mention of the rumour;
- 2 = mention of the main element of the rumour;
- 3 = warning accompanied by a mention of the main element of the rumour;
- 4 = explicit refusal to share the rumour;
- 5 = not related to the task or a meta comment such as ‘I would share the rumour’.

The second coding bore on whether participants mentioned a source at all in either of the memorization tasks:

- 0 = no source is mentioned;
- 1 = an individual source is mentioned such as ‘someone told me’, or ‘My friend's dad works at the zoo and told her’;
- 2 = no defined source is mentioned but elements suggest it comes from hearsay such as ‘I heard’.

Only the answers coded 1 are further analysed.

Finally, the third and fourth codings dealt with the specific hypotheses in hand: whether participants mention a credible source, and how many links they report. Since it was irrelevant whether participants mentioned a source in the first or second memorization task, we used a code that took the answers to both tasks into account.

Regarding the presence of a credible source, we coded for whether the source mentioned had a status justifying a privileged access to information. Thus, the credible source could be different from the one mentioned in the rumour read by the participants. For example, ‘the father of a friend’ is not a credible source but ‘a friend who works at the zoo’ is a credible source for the rumour about the bear that escaped from the zoo.

- 0 = no credible source is mentioned in either memorization tasks;
- 1 = a credible source is mentioned in at least one memorization task.

Regarding the number of links, it was coded on the basis of the answer from the memorization question that mentioned the most links:

- 1 = one link (e.g. ‘a friend’);
- 2 = two links (e.g. ‘a friend of a friend’);
- 3 = three links (e.g. ‘a friend of a friend of a friend’);
- 4 = four links (e.g. ‘a friend of a friend of a friend of a friend’).

We also coded whether the credible source, if present, was always positioned at the last link, but since the credible source was always the last link, we do not report these results further (i.e. there were no ‘a friend who is a cop has a friend who told him’; see ESM).

Twenty per cent of rumours (43 rumours) were re-coded by an independent coder blind to our hypotheses. Coders agreed on 92% of the observations (*κ* = 0.84, SE = 0.02; CI [0.80, 0.87]), and the strength of agreement is considered to be very good (Fleiss et al., 2013).

### 4.5. Model

We used evolutionary causal matrices to model the transformations occurring during transmission (here, modifications of the number of links in the source of the rumour), and to simulate the long-term effects of these transformations (Claidière et al., 2014). These models describe a situation in which a new group of participants (a new generation of agents) would receive the rumour attribution transmitted by our participants, and would behave in the same way as our participants did, in terms of how many links they mention as a function of the number of links present in the rumour they received.

The model makes the following assumptions. First, it assumes that the transmission process is similar to a Markov process in being memoryless: agents at each new generation behave exactly like those from other generations, conditional on the input they receive. Second, since we have no data on how people transmit rumours that have four links, we added the four-links answers to the three-links answers (this only affected seven answers). Third, the model assumes that the total number of participants per generation remains constant, neglecting the participants who fail to report the rumour or its source altogether. This assumption is necessary since otherwise all models would lead to the rapid extinction of the phenomenon of interest owing to the inevitability of loss in simple transmission chains (a phenomenon well known at least since Bartlett, 1932). Fourth, the model focuses on agents that mention the credible source. Since Study 2 does not provide rumours with no credible source as inputs, we cannot model what happens when agents received such rumours.

### 4.6. Results

**Figure 2.**
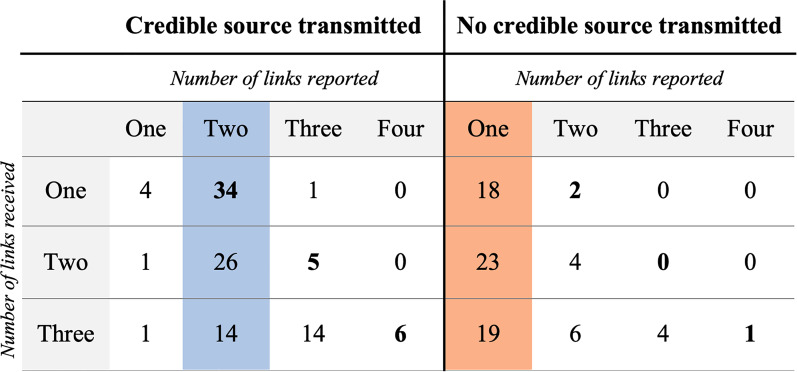
Number of links reported (*x axis*) as a function of the number of links received (*y axis*). All of the rumours received mentioned a credible source. The blue column corresponds to the dominant transmission pattern for source with a credible source (two links, e.g. a friend of a friend). The orange column corresponds to the dominant transmission pattern for source with no credible source (one link, e.g. a friend). The number in bold correspond to the number of links participants should have transmitted if they had wanted to accurately describe how they had received the information.

Out of 184 participants, 106 participants transmitted a credible source and 78 did not (Figure 2). Among rumours transmitted with a credible source, two-link attributions were more frequent than all the other links type (74 vs. 32, *χ*^2^(1, *N =* 106) = 16.64, *p* < 0.001, *φ* = 0.40). Among rumours transmitted without a credible source, one-link attributions were more frequent than all the other links type (60 vs. 17, *χ*^2^(1, *N =* 78) = 24.01, *p* < 0.001, *φ* = 0.55).

We observed a near perfect dissociation between rumours transmitted with one link, which very rarely mentioned the credible source (six out of 66, or 9.1%), and rumours transmitted with more than one link, which nearly always mentioned the credible source (99 out of 112, or 88.4%).

**Figure 3.**
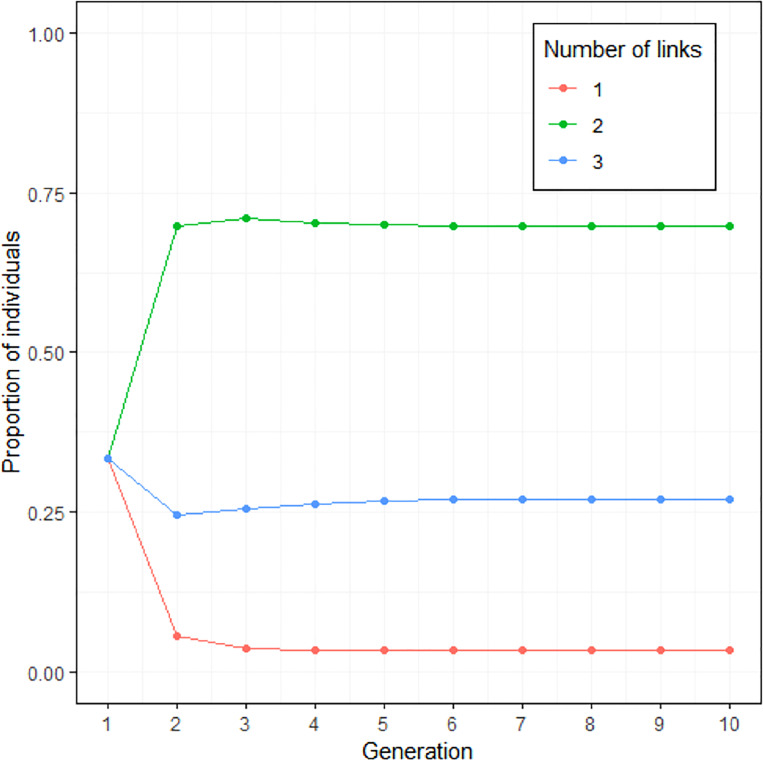
Simulation of the evolution of the number of links when a credible source is mentioned. The parameters were chosen based on the results of Study 2 (Figure 2, credible source condition with links 3 and 4 merged). Note that the model always converges towards the same equilibrium, independently of the initial proportion.

Extrapolating from the results of Figures 2 and 3 displays the outcome of the modelling. The proportion of each number of links mentioned in a constant population is modelled. At equilibrium, rumours with two links dominate in the population.

### 4.7. Discussion

The way in which participants attributed the rumours to different sources fell into a clear pattern. With a few exceptions, the participants who did not mention the credible source only reported one link, (e.g. ‘Someone just told me that a bear escaped from the zoo!’). In contrast, the participants who did mention the credible source tended to report two links, sometimes more, but very rarely one (e.g. ‘My friend told me that his dad who works at the water plant told him that the water supply is contaminated’). Of particular interest, among the participants who mentioned a credible source, most mentioned a credible friend of a friend, whether this represented an accurate attribution (when they had received a rumour attributed to a credible friend) or a shortening of what should have been an accurate attribution (e.g. participants who received a rumour attributed to a credible friend of a friend rarely attributed the rumour they passed along to a credible friend of a friend of a friend).

The results of Study 2, along with their extrapolation through modelling, suggest that as long as participants mention a credible source in the rumours they transmit, the rumour chain stabilizes at two links: the credible friend of a friend pattern. However, this is an idealization, since 42.4% of participants failed to transmit the credible source further. Because all of the rumours received by the participants in Study 2 initially contained a credible source, we cannot build a complete model of rumour transmission from these data, as we do not know how people transmit rumours they have received without a credible source. Study 2 is also unlikely to detect the potential invention of a credible source, since one was already provided. Observational evidence suggests that people often invent such credible sources in the course of rumour transmission (Bird, 1980; Blake,McFaul & Porter, 1974; De Fleur, 1962, p. 67; DiFonzo & Bordia, 2007; Kapferer, 1992; Knapp, 1944). Study 3 addresses both of these issues.

## 5. Study 3

Study 3 is similar to Study 2 but extends it in three ways. First, it introduces as input rumours attributed to non-credible sources, allowing us to construct more complete models of rumour transmission. Second, it introduces new materials – less plausible variants of the rumours from the previous studies – to test the robustness of the results from Study 2. Third, it introduces a measure of participants’ explicit willingness to share the rumours, allowing us to test whether people prefer to share: (a) rumours attributed to credible sources; (b) rumours with fewer links in their attribution; or (c) rumours with more plausible content (see, Jaeger et al., 1980; Rosnow et al., 1986; Tree & Weldon, 2007). The extent to which people are willing to share a given item is key in determining its cultural success (O. Morin, 2015), e.g. a rumour that would be easily memorized (and thus faithfully transmitted) but that no one ever wanted to share would not enjoy any cultural success.

### 5.1. Participants

A power analysis for a multiple regression with an estimated effect size of *f*^2^ = 0.15 (corresponding to a medium effect size), an *α*-level of 5% and a power of 80%, suggested that we needed a minimum of 68 participants. Since we have six conditions, we needed a minimum of 408 participants.

We recruited 800 online participants from the US, UK and Ireland, using Prolific Academic, paid £0.3. We removed 48 participants whose answer to the memorization task was not an attempt to transmit the rumour, 16 participants who explicitly refused to pass along the rumour, 26 participants who warned their best friend without transmitting the rumour (codes 1, 4, and 5 of our first coding), and 16 participants who did not provide any identifiable source (codes 0 and 2 of our second coding), leaving 694 participants (413 women, *M*_Age_ = 34.78, SD = 12.86).

### 5.2. Design and procedure

The design and procedure are similar to those in Study 2, except that the rumours presented to the participants were varied along more dimensions. Besides the number of links present in the source, we also manipulated the presence or absence of a credible source, as well as the plausibility of the rumours by creating less plausible versions of each of the rumours from Study 2. As a result, the plausible, credible source versions of the rumours in Study 3 offer a direct replication of Study 2.

The other modification to the procedure is that participants, before they answered the memorization questions on the rumour and its source, were asked how likely they would be to share the rumour with friends or relatives on a scale ranging from ‘very unlikely to share’ (1), to ‘very likely to share’ (7).

### 5.3. Materials

Here is an example of an implausible rumour about a serial killer attributed to a credible friend:

Imagine that you're doing grocery shopping and you run into a childhood friend who tells you:

‘I just learned that there's a serial killer who eats women in town. A friend told me. Be careful!’

[*Page break*]

In that situation, how likely would you be to share this information with friends or relatives?

*Likert scale: (1) Very unlikely to share – (2) – (3) – (4) – (5) – (6) – (7) Very likely to share*

[*Page break*]

Once you get home you decide to warn your best friend. You call them and tell them:

[*Free text entry*]

[*Page break*]

Your best friend says:

‘Thank you for warning me! How do you know about this again?’

What do you tell them?

[*Free text entry*]

### 5.4. Coding

The rumours transmitted by the participants, as well as the source they were attributed to when asked, were coded by one of the experimenters according to the scheme described in the coding section of Study 2.

Twenty per cent of rumours (159 rumours) were re-coded by an independent coder blind to our hypotheses. Coders agreed on 90.23% of the observations (*κ* = 0.81, SE < 0.01; CI [0.79, 0.82]); the strength of agreement is considered to be very good (Fleiss et al., 2013).

### 5.5. Results

**Figure 4.**
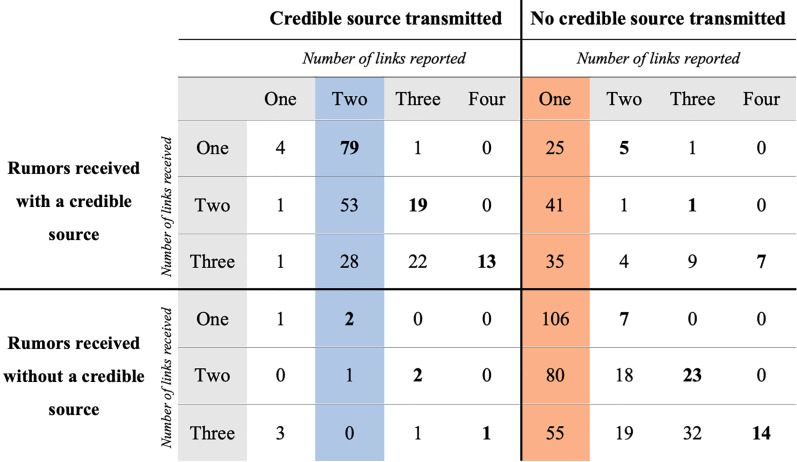
Number of links and mention of a credible source in the reported rumour (*x* axis) as a function of the number of links and mention of a credible source in the rumour received (*y* axis). The blue column corresponds to the dominant transmission pattern for rumours reported with a credible source (two links). The orange column corresponds to the dominant transmission pattern for rumours reported without a credible source (one link). The number in bold correspond to the number of links participants should have reported if they had wanted to accurately describe how they had received the information.

As in Study 2, we divided the answers in two groups: those that mentioned a credible source (*N =* 232), and those that didn't (*N =* 462) (see Figure 4).

Among rumours transmitted with a credible source, two-link sources were more frequent than all the other link types (163 vs. 69, *χ*^2^(1, *N =* 232) = 38.09, *p* < 0.001, *φ* = 0.41). Among rumours transmitted with no credible source, one-link sources were more frequent than all the other link types (342 vs. 120, *χ*^2^(1, *N =* 462) = 106.68, *p* < 0.001, *φ* = 0.48). This effect was replicated for rumours with high plausibility (among credible two-links: *χ*^2^(1, *N =* 120) = 22.53, *p* < 0.001, *φ* = 0.43; among non-credible one-link: *χ*^2^(1, *N =* 236) = 57.02, *p* < 0.001, *φ* = 0.49), and extended to rumours with low plausibility (among credible two-links: *χ*^2^(1, *N =* 112) = 15.75, *p* < 0.001, *φ* = 0.38; among non-credible one-link: *χ*^2^(1, *N =* 226) = 49.72, *p* < 0.001, *φ* = 0.47).

Among rumours received with a credible source, as in Study 2, we observed a near perfect dissociation between rumours transmitted with one link, which very rarely mentioned the credible source (six out of 107, or 5.6%), and rumours transmitted with more than one link, which nearly always mentioned the credible source (215 out of 236, or 91.5%).

These results are strengthened by the analysis of the answers to the willingness to transmit question. Among rumours transmitted with a credible source, participants were more willing to transmit those with two links (*N =* 163, *M =* 5.40, SD = 1.87) than those transmitted with other numbers of links (*N =* 69, *M =* 4.62, SD = 2.19; t (112.18) = 2.59, *p* = 0.01, CI [0.18, 1.38], *d* = 0.40). Among rumours transmitted without a credible source, participants were more willing to transmit those with one link (*N* = 343, *M* = 4.74, SD = 2.05) than those transmitted with other numbers of links (*N =* 131, *M =* 3.96, SD = 1.98; *t*(243.03) = 3.79, *p* < 0.001, CI [0.37, 1.12], *d* = 0.38).

A multiple linear regression showed that participants were more willing to share rumours with high initial plausibility (*β* = 0.31, *t*(690) = 4.17, *p* < 0.001), low of number of links (*β* = 0.15, *t*(690) = 4.14, *p* < 0.001) and a credible source (*β* = 0.34, *t*(690) = 4.67, *p* < 0.001).

Credible sources were lost in 36.5% of transmissions (127/348) and invented in 3.1% of transmissions (11/358). For example, a participant who received ‘I just learned that some people got a severe *E. coli* bacterial infection by drinking unpasteurized milk from the supermarket! A friend of a friend told me. Be careful!’, transmitted ‘Hi, be careful buying milk from the supermarket because a friend of a friend got e-Coli’.

**Figure 5.**
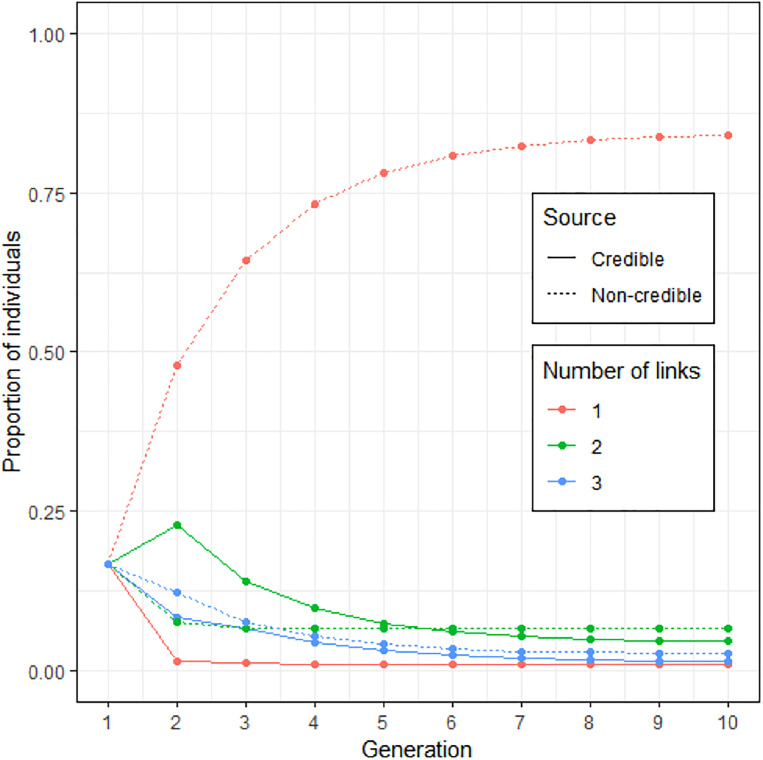
Simulation of the evolution of the number of links in conjunction to the credibility of the source (based on Figure 4, with three and four links merged). At equilibrium, about 80% of rumours in the population have a source with only one link and no credible source.

Given this asymmetry in invention and the loss rate of credible sources, a more complete version of the model used to extrapolate from the Study 2 data should reveal a rapid extinction of rumours mentioning a credible source. Figure 5 is the outcome of the modelling from Study 2 used with the data from Study 3. It displays the proportion of rumour type, as a function of number of linkd and mention of a credible source, in a constant population. At equilibrium, rumours with one link and no credible source dominate.

### 5.6. Discussion

Study 3 replicates the results of Study 2 and extends their scope by adding rumours attributed to no credible source and less plausible rumours and by measuring sharing intentions. In both studies the same pattern is observed: rumours transmitted without a credible source overwhelmingly had one link (a friend), while rumours transmitted with a credible source overwhelmingly had two links (a credible friend of a friend). However, since fewer people invent credible sources when credible sources are absent in the rumour received, than omit credible sources when they are present in the rumour received, the models show that, at equilibrium, the one-link, no credible source attribution dominates.

A limit of these models is that they approximate a situation in which each individual receives a rumour from a single individual, and passes it on to a single individual, along linear transmission chains. As mentioned above, in real life a high degree of redundancy is observed – i.e. each individual receives from and passes on the rumour to several individuals – a phenomenon that we model in Study 4.

## 6. Study 4

Modelling suggests that the attribution of rumours to a credible friend of a friend does not survive on long linear transmission chains. The model introduced in Study 4 tests whether redundancy – i.e. when each individual receives the rumour from several individuals – can allow the credible friend of a friend attribution to survive.

### 6.1. Model

**Figure 6.**
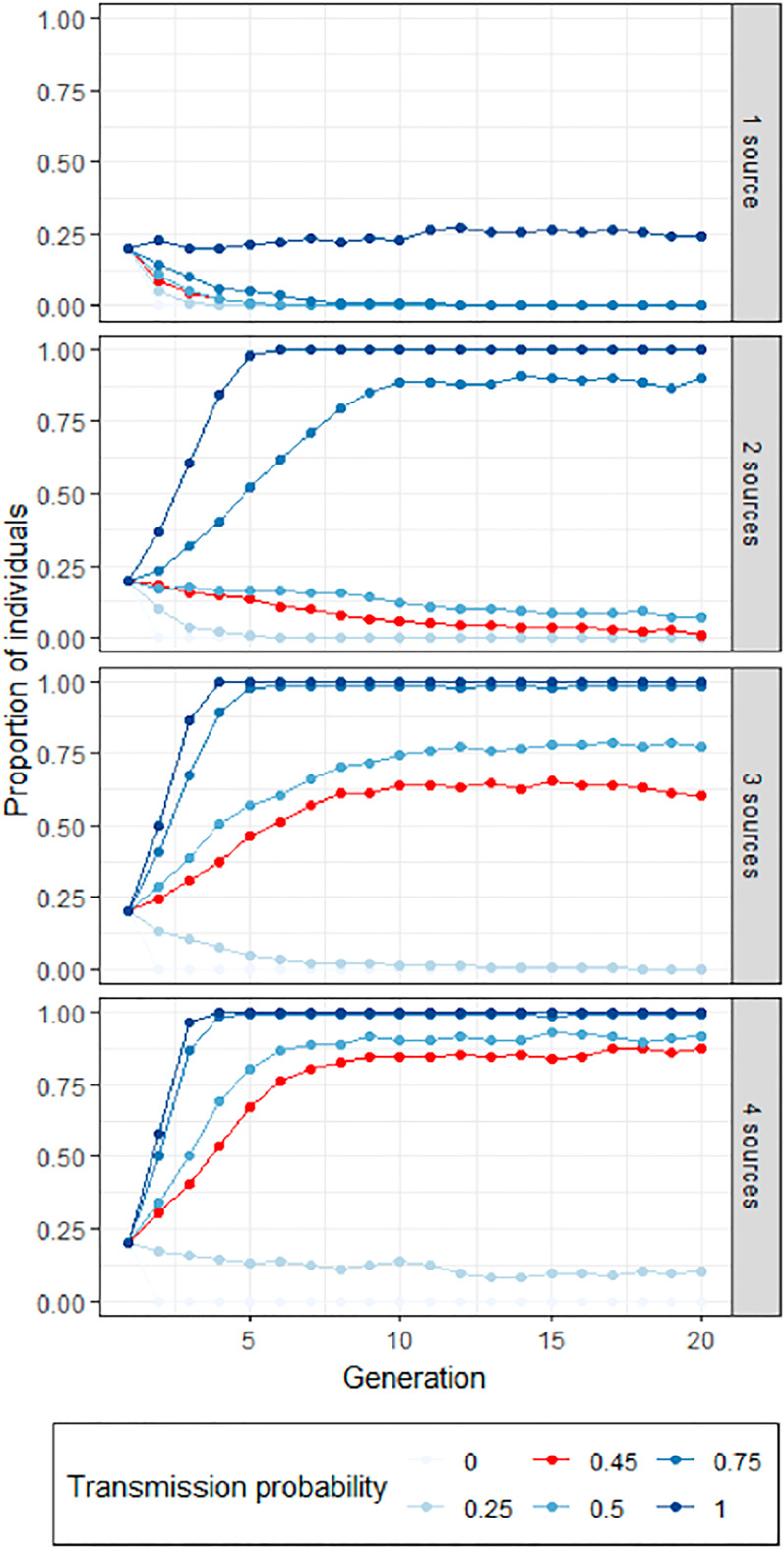
Evolution of the proportion of individuals transmitting the rumour. The number of sources has a large impact on rumour diffusion. The lines in red correspond to the values observed in Studies 2 and 3. The simulations are based on a population of 1000 individuals transmitting rumours which are either attributed to a credible friend of a friend or not. At generation 1, 20% of rumours are attributed to a credible friend of a friend.

Based on data from Studies 2 and 3, we computed the probability that someone who has received a rumour attributed to a credible friend of a friend produces a rumour also attributed to a credible friend of a friend (by dividing the number of credible friend of a friend outputs (79) by the number of credible friend of a friend inputs (175), yielding a transmission probability of 0.45). To model redundancy, we simulate a population of 1000 individuals who either have or do not have a given trait – here, attributing a rumour to a credible friend of a friend. At the first generation, 20% of the population (an arbitrary value with little impact on behaviour at equilibrium) has the trait. Then, for each new generation, each individual is exposed to one, two, three or four individuals randomly selected from the previous generation (with replacement – one individual can be exposed to the same individual from the previous generation several times). For each of these exposures, if the individual from the previous generation has the trait, they have a fixed probability of passing it on to the individual from the new generation (see Figure 6).

### 6.2. Discussion

When there is no redundancy (i.e. each individual is only exposed to one individual from the previous generation, top panel of Figure 6), the attribution to a credible friend of a friend does not spread in the population, regardless of how faithfully it is transmitted: at best, it maintains its prevalence at the first generation. In contrast, when redundancy is introduced, and each individual is exposed to several individuals from the previous generation, then the credible friend of a friend attribution can be maintained over longer transmission chains. With the transmission rate observed in Studies 1 and 2, redundancy allows the credible friend of a friend attribution to be maintained as soon as each individual is exposed to three individuals of the previous generation (Figure 6, bottom two panels).

In this model, each individual has a fixed probability of transmitting the trait to the individual from the next generation exposed to them. Alternatively, the individual from the next generation could have a fixed probability of acquiring the trait if at least one of the individual they are exposed to possesses it (see model developed in Study 5 in ESM). This form of redundancy yields overall similar results, with the credible friend of a friend attributions dominating if each individual receives the rumour from at least three sources. Overall, these results suggest that researchers should not immediately generalize from the substantial losses typically observed in linear transmission chains (see, Acerbi & Tennie, 2016; Enquist et al., 2010; Kempe et al., 2014), and that more complex but more attractive content could persevere once a measure of redundancy is taken into account.

## 7. Conclusion

Observational studies of rumours suggest that successful rumours (or related contents such as urban legends) are often attributed to a credible friend of a friend (Heath et al., 2005; Kapferer, 1992, 2013; E. Morin, 1969; Nicolini, 1989; Turner, 1987). In this article, we investigated the influence of rumour attribution, and particularly attributions to a credible friend of a friend, on rumour propagation.

To have a significant effect on rumour propagation, attributions to a credible friend of a friend must be transmitted alongside the rumour. However, when a friend says ‘a friend's father, who's a cop, said there was a serial killer in town’, an accurate attribution, when transmitting the rumour, would be ‘a friend's friend's father, who's a cop, said there was a serial killer in town’. Thus, for the credible friend of a friend attribution to be maintained, the rumour has to be shortened by one link at each new transmission episode.

In Studies 2 and 3, participants received a rumour whose attribution varied in terms of credibility of the source (credible or not) and number of links (friend, friend of a friend, friend of a friend of a friend). Participants were then asked to transmit the rumour from memory. When transmitting the rumour, a clear pattern emerged: participants systematically shortened the attribution when transmitting the rumour. Participants either failed to mention a credible source, and then only reported one link (a friend), or they mentioned a credible source, and then tended to report two links (a credible friend of a friend).

Modelling work (Studies 2–4) allowed us to extrapolate from the one transmission episode experimentally studied to longer transmission chains. When only rumours mentioning a credible source are taken into account (Study 2), the credible friend of a friend attribution clearly dominates. In contrast, when the possibility of not mentioning any credible source is introduced (Study 3), the attribution that dominates at equilibrium is the generic friend (‘a friend said …’). This last result is the logical outcome of two facts: (a) relatively few participants invent a credible source when they have not been provided with one; and (b) many participants presented with a credible source fail to mention it when transmitting the rumour. When studying linear transmission chains (i.e. one individual only passes the information to one other individual), such asymmetries leads to the extinction of most traits. However, the introduction of redundancy in the models – when each individual is exposed to rumours coming from different individuals – makes it possible for the credible friend of a friend attribution to spread in the population (Study 4).

Besides explaining the persistence of the credible friend of a friend attribution through systematic source shortening during transmission, we also show that credible friend of a friend attributions help the propagation of rumours in two ways. First, rumours attributed to a credible friend of a friend are deemed more plausible than the most salient alternatives (e.g. a friend, Studies 1 and 1′). Second, participants are also keener to transmit rumours attributed to a credible friend of a friend (Study 3).

Why do two attributions – a credible friend of a friend, or just a friend – dominate the way rumours are transmitted? We speculate that these two attributions could reflect two equilibria in a trade-off between rumour plausibility and personal accountability. On the one hand, people should be motivated to transmit rumours that appear more plausible, since an important motivation to pass along rumours is to show others that we are knowledgeable (Allport & Postman, 1947; Boyer & Parren, 2015; DiFonzo & Bordia, 2007). On the other hand, people want to minimize the reputational fallout from having transmitted a false rumour, if the rumour is ultimately revealed to have been unfounded.

If people were only maximizing the plausibility of the rumours they transmit, they could for instance attribute the rumours to a credible friend, or even to themselves. However, as pointed out above, doing so would involve a blatant lie that might be immediately detected by the audience (e.g. that one has been sick with *E. coli*, or that one knows well a policeman). As a result, when the rumour is revealed to be false, the individual who spread the rumour is also revealed as a liar, a large reputational cost. In contrast, when a rumour is attributed to a credible friend of a friend, even if the rumour is revealed to be false, each individual who transmits the rumour only engages in a minimal lie (when they shorten the attribution), and the reputational fallout should be much reduced.

Attributions to a friend make rumours less plausible than attributions to a credible friend of a friend, but they also limit the commitment of those who transmit the rumours, and thus the reputational fallout when the rumour is revealed to be false. The way people attribute rumours to different sources thus appears to reflect a general reputational tradeoff: people who attribute an idea to themselves are more rewarded, reputationally, but also suffer even more severe consequences if the idea turns out to have been wrong, or to have been misattributed (Altay et al., 2020; Altay & Mercier, 2020).

The strategic utilization of sources to engage in reputation management (Altay et al., 2020; Castelain et al., 2019; Mercier et al., 2019) could help us better understand rumour diffusion, even in today's digital environment. For example, on Twitter people who see a tweet because it has been retweeted, and then retweet the tweet themselves, appear to have done so from the original tweet, not the retweet (see, boyd et al., 2010). As a result, a tweet appears to have been found directly by a friend when in fact it had already gone through a potentially long transmission chain. This chain shortening might boost the transmission of problematic tweets – such as those with which we opened the present article – in the same way as it does with unfounded rumours transmitted orally.

## Supplementary material

For supplementary material accompanying this paper visit http://dx.doi.org/10.1017/ehs.2020.53. click here to view supplementary material

## Research transparency and reproducibility

The data associated with this manuscript can be accessed at: https://osf.io/bsp2e.
