# Supplementary material for: It happened to a friend of a friend: inaccurate source reporting in rumour diffusion
Source: Evol Hum Sci. 2020 Nov 11;2:e49. doi: 10.1017/ehs.2020.53 (PMC10427462; doi:10.1017/ehs.2020.53)
Supplement: Supplementary file 1 [file S2513843X20000535sup001.docx]

1. **MATERIALS**

**SERIAL KILLER**

***High Plausibility***

**1 link rumor:**

“I just learned that there’s a serial killer in town. My father, who is a cop investigating the case, told me. Be careful!”

**2 link rumor:**

“I just learned that there’s a serial killer in town. A friend, whose father is a cop investigating the case, told me. Be careful!”

**3 link rumor:**

“I just learned that there’s a serial killer in town. A friend of a friend, whose father is a cop investigating the case, told me. Be careful!”

**SERIAL KILLER**

***Low Plausibility***

**1 link rumor:**

“I just learned that there’s a serial killer who eats women in town. My father, who is a cop investigating the case, told me. Be careful!”

**2 link rumor:**

“I just learned that there’s a serial killer who eats women in town. A friend, whose father is a cop investigating the case, told me. Be careful!”

**3 link rumor:**

“I just learned that there’s a serial killer who eats women in town. A friend of a friend, whose father is a cop investigating the case, told me. Be careful!”

**WATER CONTAMINATION**

***High Plausibility***

**1 link rumor:**

"I just learned that the water reservoir of the city has been contaminated by a toxic leak in a factory. My father, who works at the wastewater treatment, told me. Be careful!"

**2 link rumor:**

"I just learned that the water reservoir of the city has been contaminated by a toxic leak in a factory. A friend, whose father works at the wastewater treatment, told me. Be careful!"

**3 link rumor:**

"I just learned that the water reservoir of the city has been contaminated by a toxic leak in a factory. A friend of a friend, whose father works at the wastewater treatment, told me. Be careful!"

**WATER CONTAMINATION**

***Low Plausibility***

**1 link rumor:**

"I just learned that the water reservoir of the city has been contaminated by a terrorist pouring radioactive material into it. My father, who works at the wastewater treatment, told me. Be careful!"

**2 link rumor:**

"I just learned that the water reservoir of the city has been contaminated by a terrorist pouring radioactive material into it. A friend, whose father works at the wastewater treatment, told me. Be careful!"

**3 link rumor:**

"I just learned that the water reservoir of the city has been contaminated by a terrorist pouring radioactive material into it. A friend of a friend, whose father works at the wastewater treatment, told me. Be careful!"

**ANIMALS ESCAPED FROM THE ZOO**

***High plausibility***

**1 link rumor:**

"I just learned that a bear escaped from the zoo today. My father, who is working at the zoo, told me. Be careful!"

**2 link rumor:**

"I just learned that a bear escaped from the zoo today. A friend, whose father is working at the zoo, told me. Be careful!"

**3 link rumor:**

"I just learned that a bear escaped from the zoo today. A friend of a friend, whose father is working at the zoo, told me. Be careful!"

**ANIMALS ESCAPED FROM THE ZOO**

***Low plausibility***

**1 link rumor:**

"I just learned that a herd of rhinos escaped from the zoo today. My father, who is working at the zoo, told me. Be careful!"

**2 link rumor:**

"I just learned that a herd of rhinos escaped from the zoo today. A friend, whose father is working at the zoo, told me. Be careful!"

**3 link rumor:**

"I just learned that a herd of rhinos escaped from the zoo today. A friend of a friend, whose father is working at the zoo, told me. Be careful!"

**BACTERIAL INFECTION**

***High plausibility***

**1 link rumor:**

"I just learned that some people got a severe E. coli bacterial infection by drinking unpasteurized milk from the supermarket! My father, who is working at the supermarket, told me. Be careful!"

**2 link rumor:**

"I just learned that some people got a severe E. coli bacterial infection by drinking unpasteurized milk from the supermarket! A friend, whose father is working at the supermarket, told me. Be careful!"

**3 link rumor:**

"I just learned that some people got a severe E. coli bacterial infection by drinking unpasteurized milk from the supermarket! A friend of a friend, whose father is working at the supermarket, told me. Be careful!"

**BACTERIAL INFECTION**

***Low plausibility***

**1 link rumor:**

"I just learned that some people got a severe Ebola infection by drinking coconut water imported from Africa at the supermarket! My father, who is working at the supermarket, told me. Be careful!"

**2 link rumor:**

"I just learned that some people got a severe Ebola infection by drinking coconut water imported from Africa at the supermarket! A friend, whose father is working at the supermarket, told me. Be careful!"

**3 link rumor:**

"I just learned that some people got a severe Ebola infection by drinking coconut water imported from Africa at the supermarket! A friend of a friend, whose father is working at the supermarket, told me. Be careful!"

1. **EXPERIMENT 4**

The goal of Experiment 4 was to simulate a situation in which participants would already be aware of a rumor, to see whether this would affect the rate of transmission of the credible source. This will allow us to model another form of redundancy, that is with individuals having a fixed probability of transmitting the credible friend of a friend rumor attribution if they have heard it from at least one source.

**2.1. Participants**

A power analysis for a goodness-of-fit tests with an estimated effect size of w = 0.3 (corresponding to a medium effect size) an α-level of 5% and a power of 80%, we needed 88 participants per condition. Since we have only one condition, we needed a minimum of 88 participants.

We recruited 200 online participants from the U.S., U.K., and Ireland, using Prolific Academic. Participants were paid 0.3£. We removed 13 participants whose answer to the memorization task was not an attempt to transmit the rumor, seven participants who warned their best friend without transmitting the rumor (codes 1, 4, and 5 of our first coding), and eight participants who did not provide any identifiable source (codes 0, and 2 of our second coding), leaving 172 participants (115 women, *M_Age_* = 35.48, *SD* = 12.92).

**2.2. Design and procedure**

Participants were told that over the past few days they had heard the same rumor from several people. Then, we presented participants one of the four vignettes with a credible friend of a friend, high plausibility rumor (so a sample of the rumors from the past experiments). After reading a rumor, participants were asked to write down what they would tell their best friend so as to warn them of the threat. Next, their best friend answered “Thank you for warning me! How do you know about this again?”, and participants wrote what they would have answered (as in Experiments 2 and 3).

**2.3. Materials**

Here is an example of a rumor about a bear who escaped from the zoo, attributed to a Credible source, two links:

Over the past few days, some people told you they’d heard that a bear escaped from the zoo.

Today, while shopping, you run into a childhood friend who tells you: “I just learned that a bear escaped from the zoo today. A friend, whose father is working at the zoo, told me. Be careful!”

[*Page break*]

Once you get home you decide to warn your best friend. You call them and tell them:

[*Free text entry*]

[*Page break*]

Your best friend says:

“Thank you for warning me! How do you know about this again?”

What do you tell them?

[*Free text entry*]

**2.4. Coding**

The rumors transmitted by the participants, as well as the source they were attributed to when asked, were coded by one of the experimenters according to the scheme described in Experiment 2 in the coding section.

20% of rumors (40 rumors) have been re-coded by an independent coder blind to our hypotheses. Coders agreed on 92.31% of the observations (κ = 0.85, SE = .02; 95% CI (0.81, 0.88), the strength of agreement is considered to be very good (Fleiss et al., 2013).

**2.5. Results**

The credible source was present in 65.7% of the transmissions (113/172). Among rumors memorized with no credible source, 1 link sources were more frequent than all the other links type (46 *versus* 10), chi-square goodness of fit test: χ2(1, N = 59) = 18.46, p<.001, φ = 0.56). Among rumors memorized with a credible source, 2 links sources were more frequent than all the other links type (78 *versus* 35), chi-square goodness of fit test: χ2(1, N = 113) = 16.36, p<.001, φ = 0.38).

Next, we studied rumor propagation in fixed size populations with different levels of redundancy, and different types or redundancy. In addition to the first form of redundancy reported in the manuscript (called “Multiple Independent Transmissions” in Figure S1), we model a situation in which an individual also hears a rumor from several sources, but that individual has a fixed probability of transmitting the credible friend of a friend rumor attribution if they have heard it from at least one source (we can use here use the share of credible friend of a friend rumors transmitted in Experiment 4, which mimics the situation represented by this model (0.66)). More formally, in the second model, each individual in a new generation is influenced by a group of one, two, three, or four sources randomly selected from the previous generation with replacement (i.e. the same individual could influence several participants in the following generation). If at least one of these individuals transmitted the rumor, then there was a fixed probability that the individual from the new generation would do so as well. We called this scenario “Threshold Transmission” (see Figure S1).

*
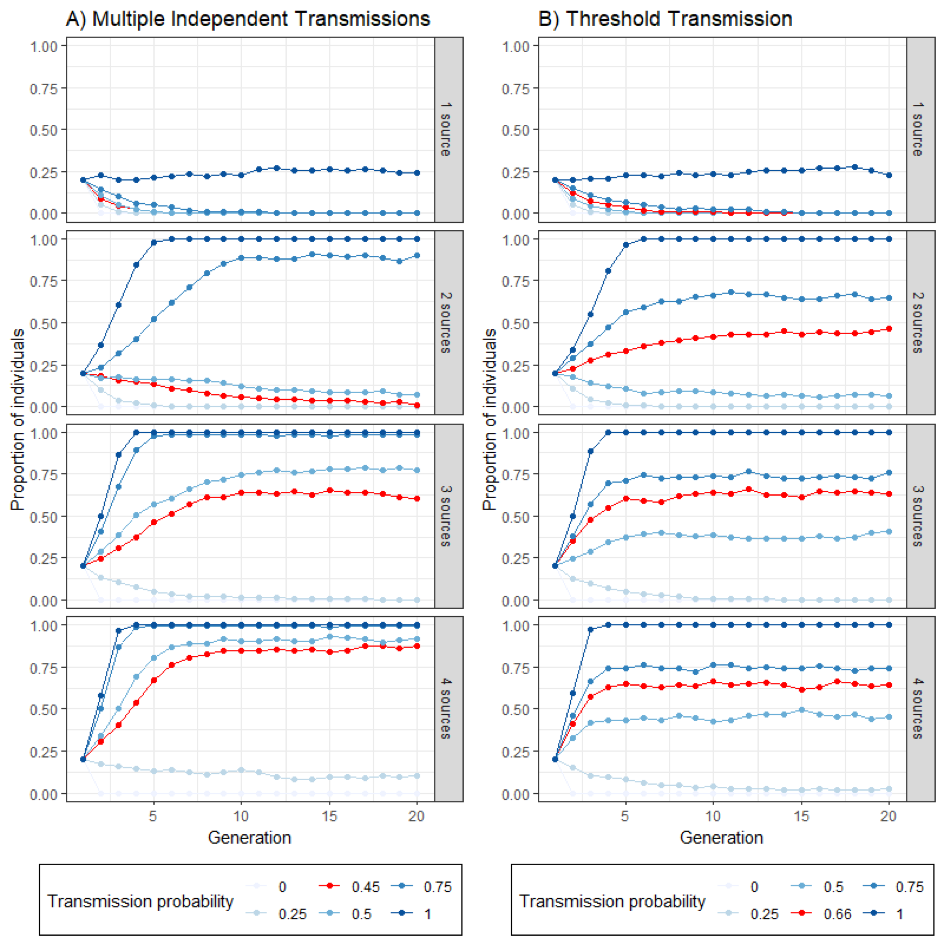
*

*Figure S1.* **Evolution of the proportion of individuals transmitting the rumor with two forms of redundancy.** The number of sources has a large impact on rumor diffusion in the two situations considered. The comparison between the Multiple Independent Transmissions model and the Threshold Transmission model also reveals that the effect of the number of sources is larger for the Multiple Independent Transmissions condition. The lines in red correspond to the reported experimental values. The simulations are based on a population of 1000 rumors with four sources (20% with a two links, credible source attribution) at generation 1.
